# Supplementary material for: Seroprevalence of Poliovirus Types 1, 2, and 3 Among Children Aged 6–11 Months: Variations Across Survey Rounds in High-Risk Areas of Pakistan
Source: Vaccines (Basel). 2025 Oct 19;13(10):1067. doi: 10.3390/vaccines13101067 (PMC12568277; doi:10.3390/vaccines13101067)
Supplement: Supplementary file 1 [file vaccines-13-01067-s001.zip › vaccines-3933318-supplementary.pdf]

Supplementary materials

Table S1. District and survey round-wise demographic information and vaccination coverage

Province	District	Survey Round	Clusters covered	Households covered	Number of children surveyed (N)	Male (%)	Vaccination Card (%)	OPV3 Coverage (%)	IPV Coverage (%)	Caregiver Education level (Illiteracy rate- %)
Punjab	Rawalpindi	1	26	327	327	51.7	93.0	95.4	97.6	19.3
		4	25	453	457	49.2	53.4	99.1	95.2	22.3
	Lahore	2	25	299	299	54.2	90.3	96.0	97.0	25.8
		4	25	487	491	48.3	56.4	79.2	59.9	29.1
	Sialkot	4	25	454	463	49.9	91.4	100.0	99.4	2.2
	Bahawalpur	4	25	445	456	50.9	86.0	98.5	95.4	68.2
	Faisalabad	4	25	436	440	48.9	78.6	97.7	89.1	30.0
	Multan	3	25	301	301	55.1	87.4	99.7	99.3	66.4
Sindh	Hyderabad	4	25	477	483	52.0	47.6	89.9	74.9	61.3
	Ghotki	4	25	451	462	53.7	42.0	56.1	43.3	73.2
	Shikarpur	4	25	477	495	52.7	14.7	72.3	29.7	0.8
	Larkana	1	25	341	341	46.0	43.4	58.4	66.3	88.6
		2	25	313	313	48.2	54.0	90.4	86.9	80.2
		3	25	303	303	48.8	84.2	88.4	90.4	84.8
		4	25	476	489	57.5	75.3	93.5	70.1	70.1
	Kashmore	4	25	467	473	51.8	18.0	69.3	27.1	93.4
	Karachi	1	75	1003	1003	52.2	72.9	72.9	78.7	38.8
		2	75	971	971	53.3	68.5	63.5	74.4	33.7
		3	75	935	936	50.6	46.0	65.7	78.7	53.1
		4	179	3171	3215	52.5	45.8	77.1	53.9	38.0
	Sukkur	1	25	300	300	50.7	44.7	44.3	61.7	75.0
		2	25	298	298	53.7	33.6	35.9	55.7	94.3
		3	25	300	300	54.7	72.3	69.3	78.0	92.0
Khyber Pakhtunkhwa	Peshawar	1	50	625	627	53.1	84.5	76.7	83.1	80.9
		2	50	650	651	49.2	91.1	83.7	89.1	82.6
		3	51	619	619	48.3	91.3	89.3	91.6	65.9
		4	25	491	494	55.9	62.1	89.5	77.1	74.1
	Khyber Agency	1	25	302	302	63.2	72.5	72.8	62.9	95.4
		2	25	300	300	59.7	69.0	99.0	100.0	94.0
		3	23	302	302	49.7	90.4	98.3	100.0	88.4
		4	25	473	475	50.3	48.6	86.7	62.5	93.7
	Bajaur Agency	4	25	457	461	48.8	81.8	90.7	69.6	89.2
	Mohmand Agency	4	25	442	461	49.5	65.3	86.6	72.5	97.0
	Kurram Agency	4	25	459	481	51.6	81.1	97.9	74.6	83.2
	Hangu	4	25	445	465	52.0	51.0	62.6	43.4	81.9
	Karak	4	25	438	453	51.2	50.1	79.7	62.5	18.5
	Kohat	4	25	434	452	50.2	69.5	83.6	67.3	57.7
	Swat	4	26	458	474	53.0	78.5	97.9	87.1	30.0
	Nowshera	4	26	475	479	51.4	72.9	88.9	69.1	60.5
	Dera Ismail Khan	4	25	496	500	46.8	34.4	88.4	76.0	88.0
	Bannu	4	25	492	495	51.7	18.8	46.3	23.4	79.8
	Lakki Marwat	4	25	471	488	52.7	19.9	56.6	34.8	90.0
	Tank	4	25	491	498	61.2	2.2	64.5	46.0	95.4

	North Waziristan	4	25	449	485	52.4	3.1	56.5	41.6	83.3
	South Waziristan	4	25	482	493	52.7	0.2	53.3	34.7	51.1
	Mardan & Swabi	1	25	314	314	56.1	85.7	89.8	93.9	63.1
		2	25	309	309	51.1	57.0	88.7	86.4	68.0
		3	25	298	298	52.7	99.0	91.3	98.3	32.6
Baluchistan	Quetta	1	25	311	311	49.8	65.6	95.5	62.7	83.9
		2	25	317	317	51.1	49.8	54.3	60.6	91.5
		3	25	310	310	61.9	60.6	31.6	75.2	72.3
		4	25	443	486	47.9	21.6	45.1	31.9	78.6
	Pishin	1	24	308	309	47.2	56.3	91.6	59.5	89.6
		2	25	310	310	50.3	49.7	44.5	50.0	96.5
		3	25	303	304	54.6	48.4	33.2	58.9	92.8
		4	25	449	464	47.8	15.7	52.8	25.9	92.2
	Killa Abdullah	1	26	312	312	50.3	34.3	55.1	26.0	98.7
		2	25	326	326	51.5	40.2	33.7	39.9	100.0
		3	25	314	314	53.5	28.3	20.7	48.1	84.1
		4	25	449	462	52.8	15.2	64.3	58.7	85.3
	Zhob	4	25	451	465	54.0	23.2	42.2	22.8	95.9
	Dera Bugti	4	26	417	437	57.0	4.1	22.7	0.9	85.4
	Nasirabad	4	25	453	460	50.9	10.0	56.1	34.1	98.5
	Jaffarabad	4	25	455	469	54.8	0.4	97.4	50.7	98.9
	Lasbela	4	27	469	480	51.5	7.3	47.9	31.7	79.6
	Mastung	4	25	408	450	49.8	3.8	69.6	49.1	74.4
	Chaman	4	25	442	459	52.1	18.3	59.0	33.6	92.8
Islamabad Capital Territory	Islamabad	4	25	467	470	54.7	51.7	95.1	87.0	23.2

Abbreviations: IPV: Inactivated Poliovirus Vaccine; OPV: Oral Poliovirus Vaccine

Table S2. Polio seroprevalence by types across provinces and survey rounds

Survey Round	Province	Poliovirus type 1 %(95% CIs)	Poliovirus type 2 %(95% CIs)	Poliovirus type 3 %(95% CIs)
1	Punjab	99.7 (99,100.3)	83.4 (79.2,87.7)	99.3 (98.4,100.3)
1	Sindh	99 (98.5,99.5)	61.7 (59.4,64.1)	95.3 (94.3,96.3)
1	KPK	98.5 (97.9,99.2)	71.6 (69,74.1)	94.9 (93.7,96.2)
1	Balochistan	91.5 (89.7,93.3)	59.8 (56.7,63)	86.5 (84.3,88.7)
2	Punjab	99.7 (99,100.3)	55.3 (49.6,61)	99 (97.8,100.1)
2	Sindh	98.9 (98.3,99.4)	52.9 (50.4,55.3)	96.1 (95.1,97)
2	KPK	98.2 (97.4,98.9)	67.7 (65.1,70.3)	96.7 (95.7,97.7)
2	Balochistan	95.8 (94.5,97.1)	78 (75.3,80.6)	92.1 (90.4,93.8)
3	Punjab	98.3 (96.8,99.8)	58.7 (53.1,64.4)	95.2 (92.8,97.7)
3	Sindh	99.2 (98.8,99.7)	34.6 (32.2,36.9)	96.7 (95.9,97.6)
3	KPK	98.6 (97.9,99.3)	78.1 (75.8,80.5)	97.3 (96.4,98.2)
3	Balochistan	98.5 (97.7,99.3)	82.1 (79.6,84.5)	96 (94.7,97.2)
4	Punjab	98.6 (98.1,99)	53.2 (51.3,55.1)	96.1 (95.4,96.9)
4	Sindh	97.3 (96.9,97.7)	43.9 (42.6,45.2)	91.6 (90.8,92.3)
4	KPK	95.8 (95.4,96.3)	57.4 (56.3,58.5)	91.1 (90.4,91.7)
4	Balochistan	89.7 (88.8,90.6)	40.4 (38.9,41.8)	81.1 (79.9,82.2)

Abbreviation: CI: Confidence Interval

Table S3. Polio seroprevalence by types across high-risk cities and survey rounds

Survey Round	District	Poliovirus type 1 %(95% CIs)	Poliovirus type 2 %(95% CIs)	Poliovirus type 3 %(95% CIs)
1	Karachi	98.9 (98.3,99.5)	65.2 (62.2,68.1)	95.5 (94.2,96.8)
1	Peshawar	98.7 (97.8,99.6)	64.6 (60.8,68.3)	95.5 (93.9,97.1)
1	Quetta	94.8 (92.3,97.3)	74.1 (69.2,79)	92.9 (90,95.8)
1	Pishin	91.9 (88.8,94.9)	44.5 (38.9,50)	86.7 (82.9,90.5)
1	Killa Abdullah	87.8 (84.2,91.5)	60.9 (55.5,66.3)	80.1 (75.7,84.6)
2	Karachi	98.9 (98.2,99.5)	42.9 (39.8,46)	96 (94.7,97.2)
2	Peshawar	98.3 (97.3,99.3)	57.9 (54.1,61.7)	97.3 (96.1,98.6)
2	Quetta	97.8 (96.2,99.4)	81.1 (76.8,85.4)	95.6 (93.3,97.8)
2	Pishin	97.7 (96,99.4)	88.6 (85,92.2)	96.1 (93.9,98.3)
2	Killa Abdullah	92.0 (89,94.9)	64.8 (59.6,70)	84.9 (81,88.8)
3	Karachi	99.1 (98.6,99.7)	38.5 (35.4,41.6)	97.6 (96.7,98.6)
3	Peshawar	99.5 (98.9,100.1)	78.9 (75.6,82.1)	97.7 (96.5,98.9)
3	Quetta	99.7 (99,100.3)	93.1 (90.2,95.9)	96.7 (94.7,98.7)
3	Pishin	98.3 (96.9,99.8)	87.4 (83.7,91.2)	97 (95.1,98.9)
3	Killa Abdullah	97.5 (95.7,99.2)	66.2 (61,71.5)	94.3 (91.7,96.8)
4	Karachi	97.5 (97,98.1)	46.8 (45.1,48.5)	93.3 (92.4,94.2)
4	Peshawar	98.7 (97.7,99.7)	69.9 (65.7,74.1)	93.9 (91.8,96.1)
4	Quetta	96.4 (94.7,98.1)	61.7 (57.3,66.1)	89.8 (87,92.5)
4	Pishin	91.7 (89.2,94.3)	41.3 (36.7,45.9)	84.6 (81.3,87.9)
4	Killa Abdullah	76.2 (72.2,80.1)	36.7 (32.3,41.2)	67.5 (63.1,71.8)

Abbreviation: CI: Confidence Interval
